# Supplementary material for: METTL14 modulates the nasopharyngeal carcinoma microenvironment via m6A-modified YWHAH identified through single-cell and machine learning analyses
Source: Front Immunol. 2026 Feb 2;17:1717039. doi: 10.3389/fimmu.2026.1717039 (PMC12907370; doi:10.3389/fimmu.2026.1717039)
Supplement: Supplementary file 6 [file DataSheet1.docx]

**
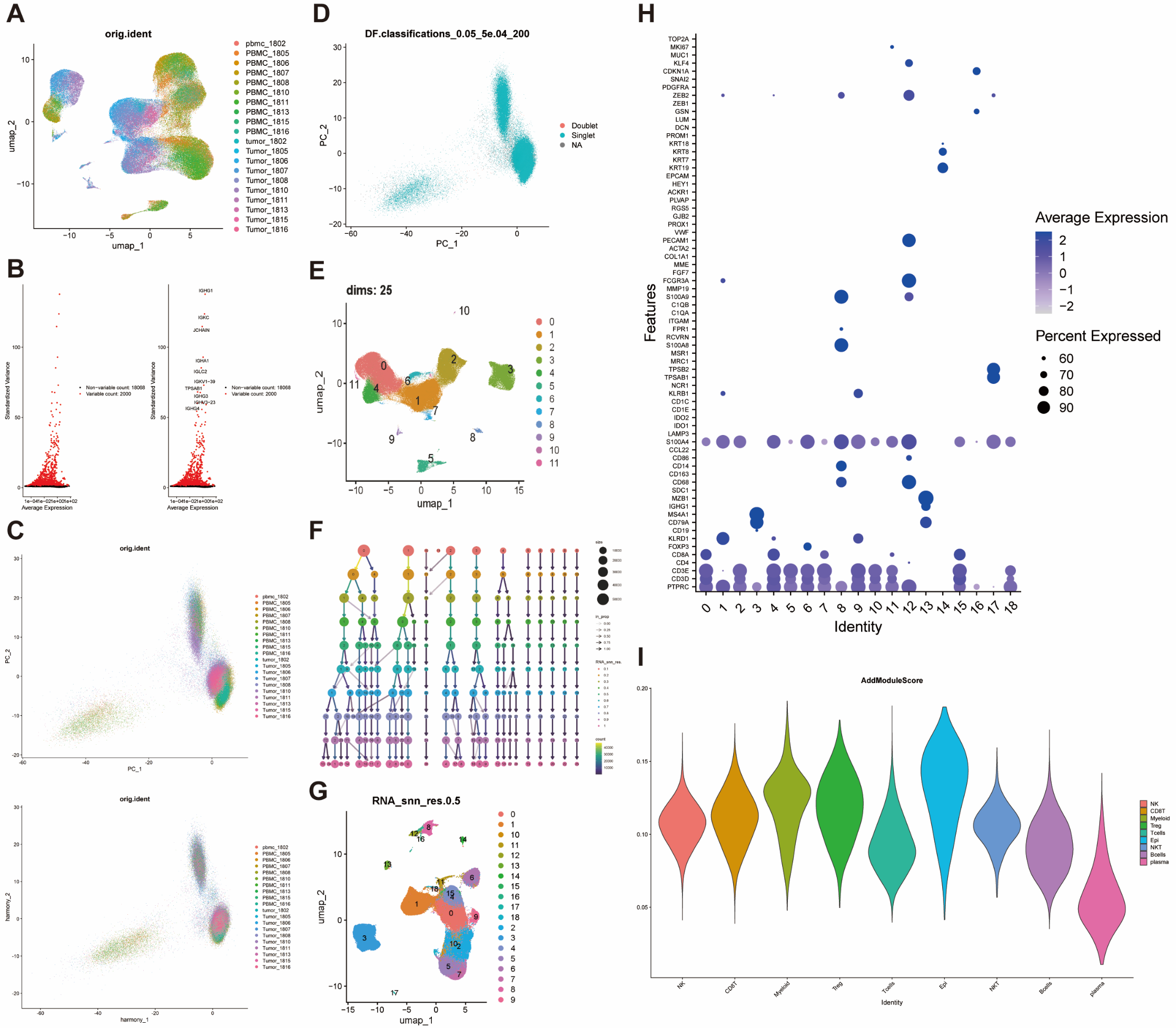
**

**Supplementary Figure 1**

(A) Original scRNA-seq data from GSE162025

(B) Identification of top 2000 highly variable features

(C) Comparison before and after PCA

(D) Identification of doublets

(E-G) Clustering of cells (dim = 25, resolution = 0.5)

(H) Cell type–specific marker genes

(I) “AddModuleScore” across different cell types


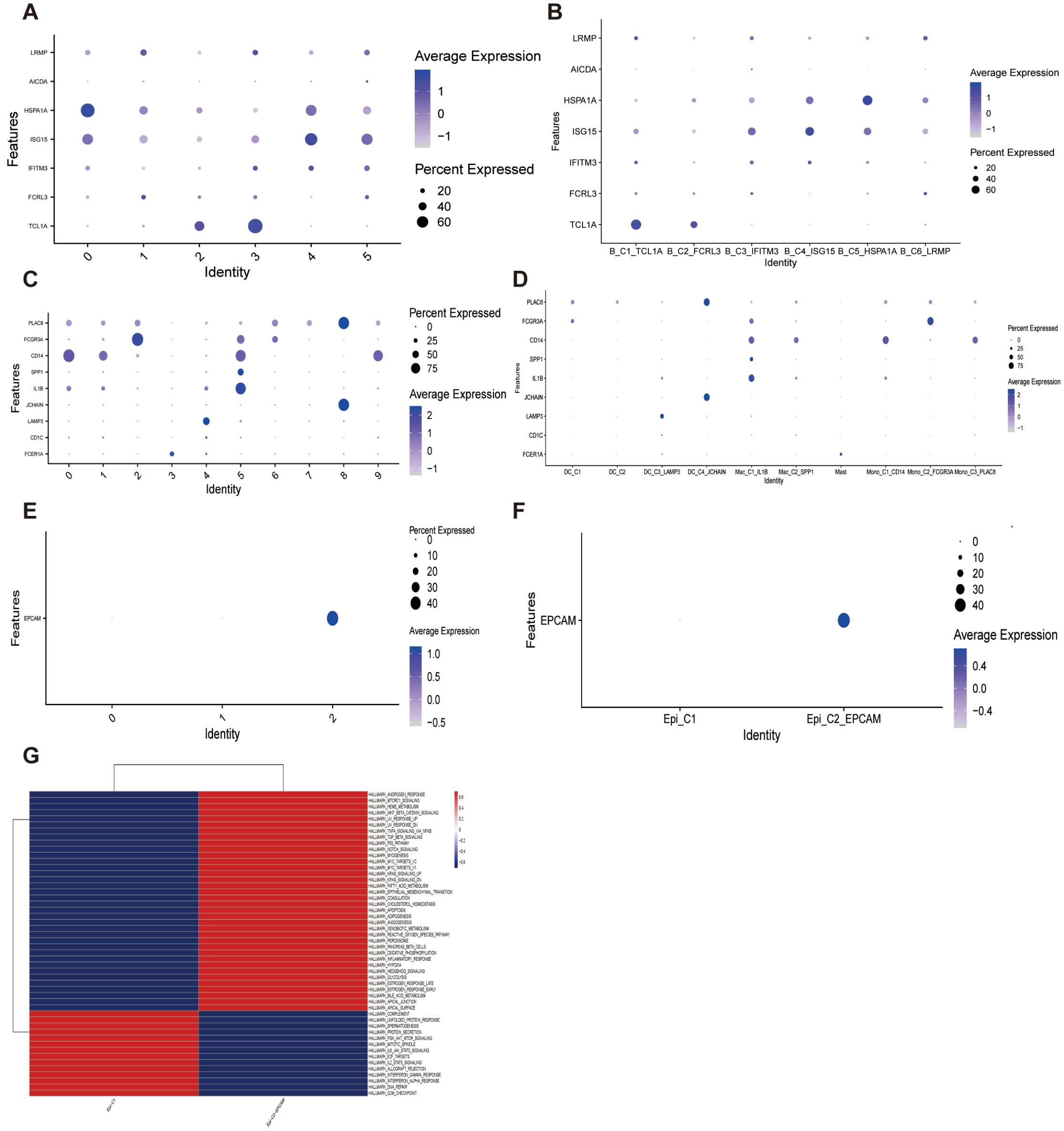


**Supplementary Figure 2**

(A-B) Specific marker genes across B cell subtypes

(C-D) Specific marker genes across myeloid cell subtypes

(E-F) Specific marker genes across epithelial cell subtypes

(G) scGSVA analysis of epithelial cells


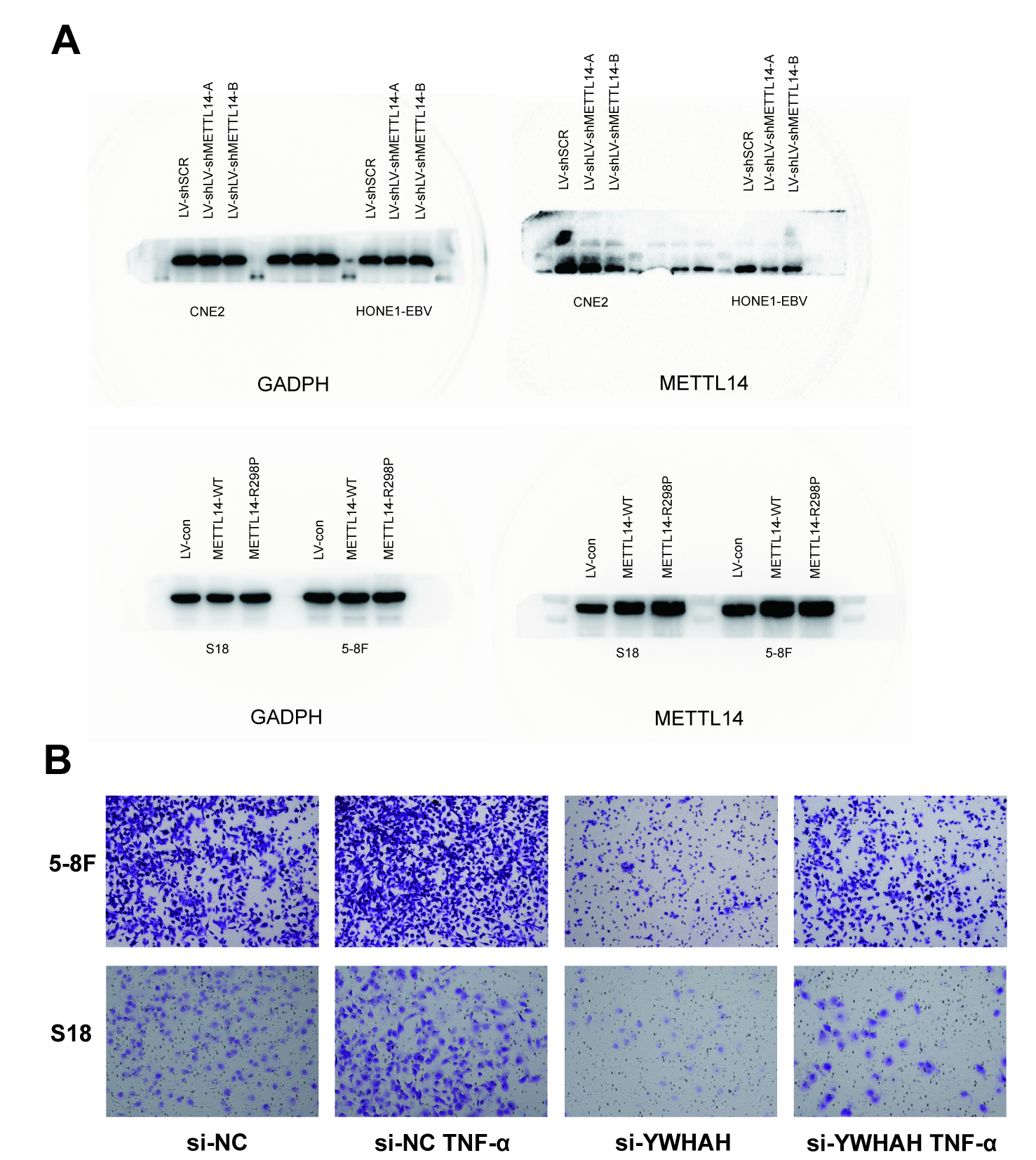


**Supplementary Figure 3**

(A) Western blot analysis of METTL14 expression in overexpression and knockdown NPC cell models.

(B) Transwell migration assays of NPC cells with YWHAH silencing and TNF-α treatment
